# Translation and Validation of the Modified A-DIVA Scale to European Portuguese: Difficult Intravenous Access Scale for Adult Patients

**DOI:** 10.3390/ijerph17207552

**Published:** 2020-10-17

**Authors:** Paulo Santos-Costa, Liliana B. Sousa, Fredericus H.J. van Loon, Anabela Salgueiro-Oliveira, Pedro Parreira, Margarida Vieira, João Graveto

**Affiliations:** 1 Health Sciences Research Unit: Nursing, Nursing School of Coimbra, 3004-011 Coimbra, Portugal; baptliliana@esenfc.pt (L.B.S.); anabela@esenfc.pt (A.S.-O.); parreira@esenfc.pt (P.P.); jgraveto@esenfc.pt (J.G.); 2 Doutorando em Enfermagem, Instituto de Ciências da Saúde da Universidade Católica Portuguesa, 4169-005 Porto, Portugal; 3 Department of Anesthesiology and Intensive Care, Catharina Hospital, 5623 EJ Eindhoven, The Netherlands; rick.v.loon@catharinaziekenhuis.nl; 4 Department of Technical and Anesthesia Nursing Sciences, Fontys University of Applied Sciences, 5631 BN Eindhoven, The Netherlands; 5 Instituto de Ciências da Saúde (Porto), Universidade Católica Portuguesa, 4169-005 Porto, Portugal; mmvieira@porto.ucp.pt

**Keywords:** peripheral catheterization, risk assessment for difficult intravenous access, scale psychometric validation

## Abstract

(1) Background: In Portugal, no accurate and reliable predictive instruments are known that could assist healthcare professionals in recognizing patients with difficult venous access. Thus, this study aimed to translate and validate the Modified A-DIVA scale to European Portuguese. (2) Methods: A methodological and cross-sectional study was conducted in two phases: translation of the Modified A-DIVA scale to European Portuguese following six stages proposed by Beaton and collaborators, and assessment of its psychometric properties in a non-probability sample of 100 patients who required peripheral intravenous catheterization in a Portuguese hospital. (3) Results: The European version of the Modified A-DIVA scale (A-DM scale) showed excellent inter-rater accordance scores, k = 0.593 (95% CI, 0.847 to 0.970), *p* < 0.0005. The A-DM scale’s criterion and construct validity was assessed through predictive, convergent, and correlational analysis with variables identified in the literature as associated with difficult peripheral intravenous access, with moderate to large magnitudes and statistical significance. (4) Conclusions: The A-DM scale is a reliable and valid instrument that can support healthcare professionals and researchers in the early identification of patients at risk of difficult peripheral intravenous access. Future validation studies are needed to test the A-DM scale’s applicability across clinical settings and in different patient cohorts.

## 1. Introduction

Obtaining peripheral intravenous access is an essential procedure in modern healthcare, contributing to the administration of medication, fluids, and contrast required for imaging [1,2]. Peripheral intravenous catheter (PIVC) insertion is frequently qualified as a nursing procedure, although this differs between international settings, which may explain the high variability of clinical practices reported in the literature [3,4,5]. In fact, while nurses are the primary PIVC inserters in countries from Europe (79%), North America (69%), or Asia (84%) [4], other countries such as New Zealand and Australia present higher numbers of PIVC insertion by doctors, technicians, or multidisciplinary specialist teams [4,5]. Regardless of their role, the main concern for healthcare professionals performing PIVC insertion is to succeed on their first attempt, without compromising care quality and patient experience, and in the shortest time possible [3].

However, PIVC insertion can be deemed a difficult procedure even for experienced healthcare professionals, compromising first-attempt success [2,3,6]. For patients experiencing difficult peripheral venous access, multiple attempts are needed to successfully insert a PIVC, resulting in a traumatic and painful experience, compromising their care experience, and potentially hindering their trust in healthcare professionals [3,7,8]. For healthcare professionals, difficulty in obtaining peripheral intravenous access is a frustrating, challenging, and time-consuming scenario, with significant associated costs and often requiring the involvement of other health professionals for subsequent attempts [3,9]. Additionally, multiple attempts are associated with complications such as extravasation and phlebitis, contributing to the depletion of the peripheral intravenous network and treatment delay.

To avoid such negative outcomes, current recommendations for good practice highlight the need to conduct a proper assessment of the patients’ peripheral intravenous network [10,11]. This fundamental step starts at the time of admission and continues as a diagnosis is established, based on the assessment of the patient’s history of difficult intravenous peripheral access, comorbidities, contraindications, available veins, and diagnosis [10]. To overcome the absence of a common approach in the assessment of the patients’ peripheral intravenous network, healthcare professionals should implement a uniform set of criteria in this field [12,13]. 

In the literature, the Modified A-DIVA Scale is highlighted as a reliable and generalizable predictive scale used by healthcare professionals to identify patients at risk of difficult intravenous access [12,14]. Deriving from the initial A-DIVA scale (developed in a cohort of surgical patients) [15], the Modified A-DIVA scale can be applied across clinical settings and patient cohorts basing its assessment on five items: (i) the patient’s history of difficult intravenous access; (ii) expected difficult intravenous access by the healthcare professional prior to an attempt at PIVC insertion; (iii–iv) the inability to detect a dilated vein by palpation and/or visualization; and (v) the diameter of the target vein is less than 3 millimeters [14]. Each confirmed item adds one point to the scale’s score (ranging from 0 to 5), where a higher score indicates a higher risk of difficult intravenous access and risk of failed PIVC insertion [14]. 

To the best of the authors’ knowledge, there are no known validated tools used in the early assessment of patients’ peripheral intravenous access in Portugal. This may potentially explain why current studies conducted in Portugal report between two to eight puncture attempts before a successful PIVC insertion in almost a quarter of the study population [16,17,18]. In fact, if considering the entire period of hospital admission, one study highlighted that an average of five puncture attempts are performed before a successful PIVC insertion, ranging between 1 and 20 puncture attempts [16]. Instruments like the Modified A-DIVA scale could assist healthcare professionals in Portugal to prospectively identify patients at risk of difficult intravenous access based on easily available clinical data [15], adjusting their approach to PIVC insertion and preserving patients’ peripheral intravenous network. Therefore, this study focused on the translation and validation of the Modified A-DIVA Scale to European Portuguese.

## 2. Materials and Methods

A methodological and cross-sectional study was conducted to analyze the validity and reliability of the Modified A-DIVA Scale after its translation to European Portuguese.

### 2.1. Phase 1—Translation of the Modified A-DIVA Scale

**Scheme 1:**
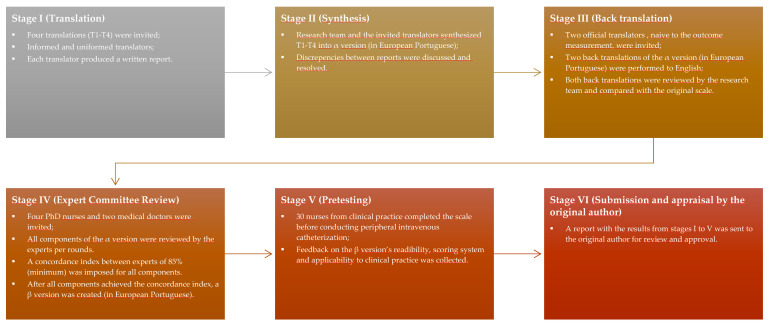
Overview of the translation process adapted from Beaton and collaborators’ guidelines [19].

The first phase was conducted according to Beaton and collaborators’ guidelines for the process of cross-cultural adaption of self-report measures [19], comprising of six stages (Scheme 1).

In stage I (Translation), reviewers with a background in nursing (*n* = 2), psychology and psychometrics (*n* = 1), and biomedical laboratory sciences (*n* = 1) were invited to independently assess and translate the Modified A-DIVA Scale to European Portuguese. All the invited reviewers were fluent in written and spoken English and had integrated the language in their professional activities, with a high knowledge of scientific and technical terms. The research team and the reviewers analyzed and discussed the four resulting translations, which were synthesized, and resulted in the development of an α version in European Portuguese (stage II). In stage III, two official translators whose native language was English back-translated the α version from European Portuguese to English. Both back-translations were reviewed by the research team in collaboration with the translators. The research team deemed that the original Modified A-DIVA Scale and the developed α version of the scale in European Portuguese had linguistic equivalence. 

To proceed with the translation process, an Expert Committee was formed (stage IV). Four PhD nurses and two medical doctors with experience in vascular access were invited to assess the α version of the scale in European Portuguese, using a standardized scoring sheet created by the research team. The experts’ agreement with each component of the scale (e.g., title, overall scale description, and items) was assessed through a Likert scale ranging from 1 (Totally Disagree) to 7 (Totally Agree), where reasoning for each score could be provided. The scale components were deemed satisfactory if a concordance index of 85% was achieved between experts. During the first round of consensus, minor linguistic changes were recommended, with all of the α version’s components achieving the required concordance threshold. One expert suggested that the title of the scale in European Portuguese should be as close to the original as possible, given that the term “DIVA patient” is widely recognized and used in the literature to classify a patient with difficult peripheral intravenous access. The experts’ contributions were incorporated by the research team, and the proposed title was Escala A-DIVA Modificada (A-DM scale). During a second round, no further recommendations were made, and all the experts deemed the scale as a valuable contribution to clinical practice and research in the field of vascular access. 

In stage V (Pretest), between November 2019 and December 2020, nurses (*n* = 30) from a surgical ward in central Portugal were requested to score the A-DM scale before inserting a PIVC. In general, the nurses considered that the items of the A-DM scale were clearly and easily scored, alluding to the fact that no deviations from normal practice were needed to answer the scale. The results gathered from the previous phases were synthesized and presented to the original author of the Modified A-DIVA Scale, who approved the final version (stage VI). 

### 2.2. Phase 2—Psychometric Validation of the A-DM Scale

The psychometric validation of the A-DM scale was conducted between December 2019 and July 2020, in a surgical ward from an oncology hospital in Portugal. The study description and objectives were presented to the nursing team by the lead researcher, who also explained how all ethical assumptions in clinical research would be complied with. All participating nurses in this study had a minimum of one year in clinical practice and previous experience in PIVC insertion; voluntary and informed consent of participation was obtained before study commencement. 

Patient recruitment followed a non-probability, consecutive sampling technique until the required sample size was achieved. Patients that were selected had to be over 18 years of age, able to make informed consent, and planned to return to the same ward after surgery. Patients with peripheral venous system damage, known intravenous drug addiction, and patients that would be transferred to another unit after the planned surgical procedure were excluded from the study. 

Two nurses assessed each patient’s peripheral venous network before PIVC insertion at the same time. A tourniquet was first applied 5–10 cm above the antecubital fossa followed by the observation and palpation of vein trajectories. After this, both nurses scored the A-DM scale independently without discussing their rationale. The nurse responsible for the patient inserted a short PIVC into the selected vein. Using van Loon and collaborators’ definition [14], a puncture attempt was defined as a percutaneous needle puncture, irrespective of subcutaneous progression. Likewise, intravenous catheterization was considered successful if the practitioner was able to inject a 0.9% sodium chloride flush without signs of infiltration. 

Several variables were collected throughout the study, including patient-related variables (e.g., age, gender, visible vein diameter in millimeters, and body mass index), procedure-related outcomes (e.g., PIVC caliber, time for catheter insertion in minutes, first-attempt success, number of puncture attempts, and complications), and nurse-reported ease of puncture (in Likert scale format, between 1—“not at all difficult” and 7—“extremely difficult”). Moreover, each nurse also scored the Venous International Assessment (VIA) scale [20] which is also undergoing a translation and validation process to the Portuguese population (hereby referred to as ENAV scale). The ENAV is a “performance status tool” [20] that allows healthcare professionals to classify the patient’s peripheral intravenous access in five grades based on three comprehensive parameters: (i) the number of observable puncture points; (ii) PIVC size and ease of performing venipuncture; and (iii) risk of extravasation or phlebitis [20]. The risk of difficult intravenous access progressively increases in accordance to each grade.

Descriptive analysis was performed to provide an overview of patient and PIVC-related variables. The A-DM’s reliability was calculated through inter-rater agreement using Cohen’s Kappa coefficient (k) [21]. The scale’s criterion and construct validity were assessed according to Boateng and associates’ recommendations [22]. Pearson’s correlation coefficient (r) was used for continuous variables, while the point biserial correlation coefficient (rpb) was used for independent dichotomous variables. A 5% level of significance (*α* = 0.05) was determined for all necessary analysis. Data analysis was performed using the Statistical Package for the Social Sciences (IBM SPSS Statistics for Windows, Version 25.0. IBM Corp.: Armonk, NY, USA). 

This study was approved by the hospital’s board after a favorable review by its Ethics Committee (ref. TI 24/2019).

## 3. Results

During phase one, the translation process of the Modified A-DIVA scale to European Portuguese showed satisfactory results, with all components of the A-DM scale achieving a concordance index of 85% between experts. The final version of the A-DM scale in European Portuguese can be found in Table 1.

During phase two, 100 PIVC insertions were observed, and the respective patient demographic and clinical variables can be found in Table 2. Overall, 83% of the patients required a PIVC insertion due to an impending surgery, while 12% had a previous non-functioning catheter. Nurses mainly opted for the veins in the back of the hand (59%) and forearm (32%), selecting mostly 20G PIVCs (79%). Successful PIVC insertion was achieved after an average of 1.57 attempts (1–8, SD ± 1.1). PIVC remained in situ for 2.1 days (0–8, SD ± 1.4). Throughout the study, a complication rate of 26% was recorded, mainly due to infiltration (18%) and phlebitis (9%).

The A-DM scores were distributed asymmetrically, with a mean score of 0.970 (0–5, SD ± 1.19). Regarding inter-rater reliability, Cohen’s k was run to determine if there was an agreement between two nurses’ judgement on the patient’s A-DM scale score (Table 3). There was an almost perfect agreement between the two nurses’ judgements, k = 0.593 (95% CI, 0.847 to 0.970), *p* < 0.0005 [21]. All Cohen’s Kappa values exceed the value of 0.800, with the reported *p*-value being constantly lower than 0.001, which supposes a considerable statistical significance [21]. 

To assess the A-DM’s criteria and construct validity, the total scores obtained were correlated with patient and procedural-related variables that are identified in the literature as hypothetically associated with intravenous access difficulty (Table 4) [2,3,6,23,24]. 

A point-biserial correlation was run to determine the relationship between the A-DM and ENAV scales’ scores. There was a positive correlation between both scales, which was statistically significant (*r*_pb_ = 0.739, *p* < 001).

## 4. Discussion

During phase one, the involvement of contributors and experts from different scientific backgrounds was deemed extremely significant since the A-DM scale is expected to be used by healthcare professionals and researchers from different areas and sites. Similarly, the involvement of two official English-Portuguese translators during stage III ensured that the α version of the A-DM scale had semantic and idiomatic equivalence to the original Modified A-DIVA Scale.

Strict compliance with the six stages proposed by Beaton and colleagues [19] resulted in the development of a translated scale for the Portuguese population. At the end of phase one, the high agreement between experts (≥85%) and positive feedback provided by the clinical practice nurses involved were valuable indicators of the A-DM scale’s potential applicability and significance to clinical practice.

According to Boateng and collaborators [22], scale validity can be assessed through criterion (predictive and concurrent) and construct validity (convergent, discriminant, differentiation by “known” groups, and correlation analysis). First, to assess criterion validity, the A-DM’s ability to predict PIVC survivability and complications was tested. The A-DM correlated positively with PIVC premature removal (r_pb_ = 0.336, *p* < 0.001). Concerning PIVC-related complications, although correlation with specific common complications in surgical settings was not statistically significant (e.g., phlebitis and infiltration), the A-DM was successful in predicting the overall development of PIVC-related complications with a magnitude of 0.157 and significant at 0.05. Given that no “gold standard” is internationally recognized in this field [12], concurrent validity could not be assessed.

Construct validity was assessed through convergent and correlational analysis. Convergent validity was assessed using the ENAV scale since it aims to measure the same latent construct as the A-DM scale [14,20]. Both scales correlated significantly with a magnitude of 0.739 (*p* < 0.001), attesting to its convergent validity.

Several correlation analyses were conducted to further quantify the A-DM’s validity [22], using a comprehensive list of patient and procedural-related variables described in the literature as potentially associated with difficult peripheral intravenous access [2,3,6,23,24]. Regarding patient-related variables, the A-DM total scores correlated significantly with patient’s age, body mass index, Type-2 Diabetes diagnosis, and previous antineoplastic treatment (such as chemo or radiotherapy). Attesting to its validity, the A-DM also correlated with the diameter of the patients’ veins (r = −523, *p* < 0.001), which is the focus of the A-DM scale’s fifth item and extensively regarded in the literature as a crucial variable in PIVC first-attempt success.

Regarding procedural variables, the A-DM correlated significantly with the caliber (gauge) of the PIVC selected by the nurses, with a magnitude of 0.517 (*p* < 0.001). Moreover, the A-DM correlated significantly with first-attempt success and the number of puncture attempts needed until PIVC insertion, both of which are intrinsically associated with the latent construct of the scale given that difficult peripheral intravenous access can often lead to lower first-attempt success rates and a higher number of puncture attempts. Furthermore, nurse-reported ease of puncture also correlated significantly with the A-DM scale scores with a magnitude of 0.620 (*p* < 0.001), attesting to its ability to measure difficult peripheral intravenous access during healthcare professionals’ assessment of the patients’ venous network. Although a comprehensive list of risk factors was retrieved from the literature and used to test the A-DM’s validity, there is no international consensus on which factors are associated with higher chances of difficult intravenous access [25,26]. This may explain why some of the included patient and procedural variables did not correlate significantly with the A-DM scale (e.g., patient gender or PIVC insertion locations).

According to Boateng and collaborators [22], the A-DM’s validity is supported if at least two forms of construct validity have been assessed. In this study, satisfactory results were achieved through predictive, convergent, and correlational analysis. However, study limitations must be addressed, such as the non-probability, consecutive sampling technique used to recruit participants from a specific clinical setting. Further validation studies in different clinical sites and involving specific patient cohorts are needed to test the A-DM’s transversal applicability. Future studies should test the A-DM’s predictive nature with more comprehensive study samples, allowing for the analysis of its functioning in individuals that display hypothetical risk factors for difficult peripheral intravenous access (e.g., gender). While this study was one of the first to also assess PIVCs’ survival times after an initial assessment with a scale [12], larger studies are needed to further explore the A-DM’s predictive nature concerning specific PIVC-related complications. Although not consensual, current evidence suggests that variables associated with health professionals (e.g., professional experience, professional role, and experience in PIVC insertion) are likely associated with first-attempt success and diagnosis of difficult peripheral intravenous access [24,27,28,29]. Thus, future validation studies should analyze the association between such variables and the A-DM’s scores. Likewise, future studies should identify recommendations to clinical practice following the A-DM’s score [12].

Nevertheless, the A-DM can be considered a reliable and valid contribution to clinical practice in Portugal by standardizing the initial assessment of the patient’s peripheral venous network and reporting it with a common method [10]. Early assessment of difficult peripheral intravenous access can likely decrease known high rates of the first-attempt failure and need for consequent attempts [17,18], reducing the incidence of PIVC-related complications and premature removal [30]. Although nurses are the health professionals primarily responsible for PIVC insertion and management in Portugal [31], we believe that the A-DM scale can assist any health professional with competences in vascular access by improving early assessment of the patient’s peripheral venous network and identification of difficult peripheral intravenous access, enhancing PIVC-related care quality, continuity, and record keeping [12,14].

## 5. Conclusions

The A-DM scale demonstrated semantic equivalence to the original Modified A-DIVA scale, receiving positive reactions and acceptance from the experts, clinical practice nurses, and the original author of the scale. The A-DM scale evidenced respectable reliability and validity properties, sustaining its likely contribution to clinical practice by assisting Portuguese healthcare professionals in the assessment of patients’ peripheral intravenous network. Future validation studies should be developed across clinical settings and patient cohorts, also exploring the role of professional-related variables in the scoring of the A-DM scale (e.g., professional experience and professional role).

## Figures and Tables

**Table 1:** The original and proposed version of the Modified A-DIVA scale (escala A-DIVA Modificada in European Portuguese).

Original Version (In English)	European Portuguese Version
Factor	Fator
Score	Pontuação
Is there a known history of a difficult intravenous access?	A pessoa apresenta história prévia de acesso endovenoso periférico considerado difícil?
Do you expect a failed first attempt or a difficult intravenous access?	É esperada uma primeira tentativa de punção sem sucesso ou que o acesso endovenoso periférico seja difícil?
Is there an inability to identify a dilated vein by palpating the upper extremity?	É incapaz de identificar uma veia dilatada após observação de um membro superior?
Is there an inability to identify a dilated vein by visualizing the upper extremity?	É incapaz de identificar uma veia dilatada após palpação de um membro superior?
Has the largest dilated vein a diameter less than 3 millimeters?	A veia dilatada de maior calibre apresenta um diâmetro menor que 3 milímetros?

**Table 2:** Demographic and clinical characteristics of the study participants (*n* = 100)—Phase 2.

Demographic and Clinical Variables	Patients’ Characteristics (*n* = 100)
**Age**	63.4 years (28–92; SD ± 14.2)
**Sex**	
Male	8%
Female	92%
**Comorbidities**	
Type 2 Diabetes Mellitus	16%
Arterial Hypertension	44%
Dyslipidemia	16%
**Smoker**	8%
**Previous cancer treatment**	
Chemotherapy	24%
Radiotherapy	2%
Hormone Therapy	2%
None	74%
**Body Mass Index (kg/m^2^)**	
Below 18.5	5%
18.5–24.9	26%
25.0–29.9	49%
30.0 and above	17%
*Missing*	3%

SD—Standard Deviation.

**Table 3:** Results of the inter-rater reliability analysis performed for the escala A-DIVA Modificada (*n* = 200).

Item	Cohen’s Kappa	*p*-Value
A pessoa apresenta história prévia de acesso endovenoso periférico considerado difícil?	0.917	<0.001
É esperada uma primeira tentativa de punção sem sucesso ou que o acesso endovenoso periférico seja difícil?	0.847	<0.001
É incapaz de identificar uma veia dilatada após palpação de um membro superior?	0.864	<0.001
É incapaz de identificar uma veia dilatada após observação de um membro superior?	0.847	<0.001
A veia dilatada de maior calibre apresenta um diâmetro menor que 3 milímetros?	0.970	<0.001
Total	**0.910**	**<0.001**

**Table 4:** Correlation matrix for the A-DM scale (*n* = 200).

Variables		*r/r*_pb_	*p*-Value
**Patient-related variables**	Age	0.154	0.030
	Gender	0.069	0.330
	Body Mass Index	0.151	0.033
	Visible vein diameter (in millimeters)	−0.523	<0.001
	Previous antineoplastic treatment	0.157	0.027
	Comorbidities: Arterial Hypertension	0.072	0.309
	Comorbidities: Dyslipidemia	0.011	0.879
	Comorbidities: Type-2 Diabetes	0.226	0.001
**Procedural variables**	Caliber (Gauge)	0.517	<0.001
	Insertion site: hand	−0.063	0.375
	Insertion site: forearm	0.027	0.708
	Insertion site: antecubital fossa	0.072	0.311
	First-attempt success	0.512	<0.001
	Number of puncture attempts	0.414	<0.001
	Time for PIVC insertion (in minutes)	−0.016	0.825
	Easiness of puncture	0.620	<0.001
**PIVC outcomes**	Premature removal	0.336	<0.001
	Complication: infiltration	0.086	0.226
	Complication: phlebitis	0.122	0.085
	Complications (overall)	0.157	0.027

r—Pearson correlation; *r*_pb_—point biserial correlation coefficient.
